# A multi-omics longitudinal study of the murine retinal response to chronic low-dose irradiation and simulated microgravity

**DOI:** 10.1038/s41598-022-19360-9

**Published:** 2022-10-07

**Authors:** Prachi Kothiyal, Greg Eley, Hari Ilangovan, Katherine A. Hoadley, S. Robin Elgart, Xiao W. Mao, Parastou Eslami

**Affiliations:** 1 Scimentis LLC, Statham, GA 30666 USA; 2 Science Applications International Corporation (SAIC), Reston, VA 20190 USA; 3 Department of Genetics, Lineberger Comprehensive Cancer Center, University of North Carolina at Chapel Hill, Chapel Hill, NC 27599 USA; 4 University of Houston, Houston, TX 77204 USA; 5 Basic Sciences, Loma Linda University, Loma Linda, CA 92350 USA; 6 Universal Artificial Intelligence Inc., Boston, MA 02130 USA

**Keywords:** Retinal diseases, Epigenomics, Transcriptomics

## Abstract

The space environment includes unique hazards like radiation and microgravity which can adversely affect biological systems. We assessed a multi-omics NASA GeneLab dataset where mice were hindlimb unloaded and/or gamma irradiated for 21 days followed by retinal analysis at 7 days, 1 month or 4 months post-exposure. We compared time-matched epigenomic and transcriptomic retinal profiles resulting in a total of 4178 differentially methylated loci or regions, and 457 differentially expressed genes. Highest correlation in methylation difference was seen across different conditions at the same time point. Nucleotide metabolism biological processes were enriched in all groups with activation at 1 month and suppression at 7 days and 4 months. Genes and processes related to Notch and Wnt signaling showed alterations 4 months post-exposure. A total of 23 genes showed significant changes in methylation and expression compared to unexposed controls, including genes involved in retinal function and inflammatory response. This multi-omics analysis interrogates the epigenomic and transcriptomic impacts of radiation and hindlimb unloading on the retina in isolation and in combination and highlights important molecular mechanisms at different post-exposure stages.

## Introduction

Spaceflight hazards such as microgravity and space radiation are anticipated to impact biological systems and could potentially affect crew health and performance both in mission and once astronauts return to Earth. These unique hazards have been thought to induce adverse changes in the central nervous system (CNS), with a frequently reported impact on ocular function^1–4^. Resources aboard space assets that have a sustained human presence like the International Space Station (ISS) are limited and it is difficult to recreate spaceflight conditions on the ground for controlled experiments. Therefore, characterization of the type and magnitude of these changes is challenging. Despite these challenges, analogs can be used to inform the understanding of the spaceflight environment impacts on biology and support development of mitigation strategies to reduce spaceflight health risks. Ground-based analogs can also support the interrogation of biological effects from disparate stressors in isolation from one another that cannot be untangled in the spaceflight environment. Furthermore, advances in bioinformatics have expanded the potential scientific return of prior experiments provided data is accessible for re-evaluation. Fortunately, NASA’s GeneLab is an open-access omics database for space-relevant biological experiments that provides access to uniformly formatted data^5^.

The current study leverages transcriptomic and methylation sequencing data acquired as part of a larger experiment to understand the longitudinal effects and underlying mechanisms of low-dose ionizing radiation (IR) exposure and hindlimb unloading (HLU; an analog for microgravity) on neurovascular oxidative stress and brain function^6^. Global transcriptome profiling with RNA-seq quantifies gene expression at the time of sample acquisition while reduced representation bisulfite sequencing (RRBS) analyzes DNA methylation, which is one of the most studied epigenetic modifications involved in disease development^7–9^.

Previous studies analyzed spaceflight-induced transcriptomic and epigenomic response in murine retina^10,11^ or evaluated response to different ground-based hazard analogs in murine brain^6^ and spleen^12^. The spaceflight studies reported expression and methylation changes in genes known to be involved in visual perception, phototransduction, and macular degeneration pathways in murine models. However, these studies were performed in space where effects of both space radiation and microgravity on the retina were intertwined. In addition, these studies were performed 35–37 days after a spaceflight and there was no analysis done on longitudinal changes in the retina due to radiation or microgravity.

To our knowledge, the current analysis is the first multi-omics study of longitudinal changes in the murine retina due to microgravity, low-dose radiation, or combined exposure to both. We report differentially expressed genes (DEG), differentially methylated genes (DMG), and biological processes (BP) that are exclusive to, or shared across, multiple exposure conditions and post-exposure time points. We observed similar epigenetic and transcriptomic discordance in genes reported in the NASA Twins Study^13^ and in the retina of mice flown to space^11^, with some changes persistent even 4 months after exposure. This study provides an insight into the retinal response to exposures that are frequently used as analogs for individual spaceflight hazard and their interplay at different post-exposure stages, and contributes towards identifying key pathways to interrogate that may provide translational relevance across exposure types.

## Results

### Differential gene expression

**Figure 1:**
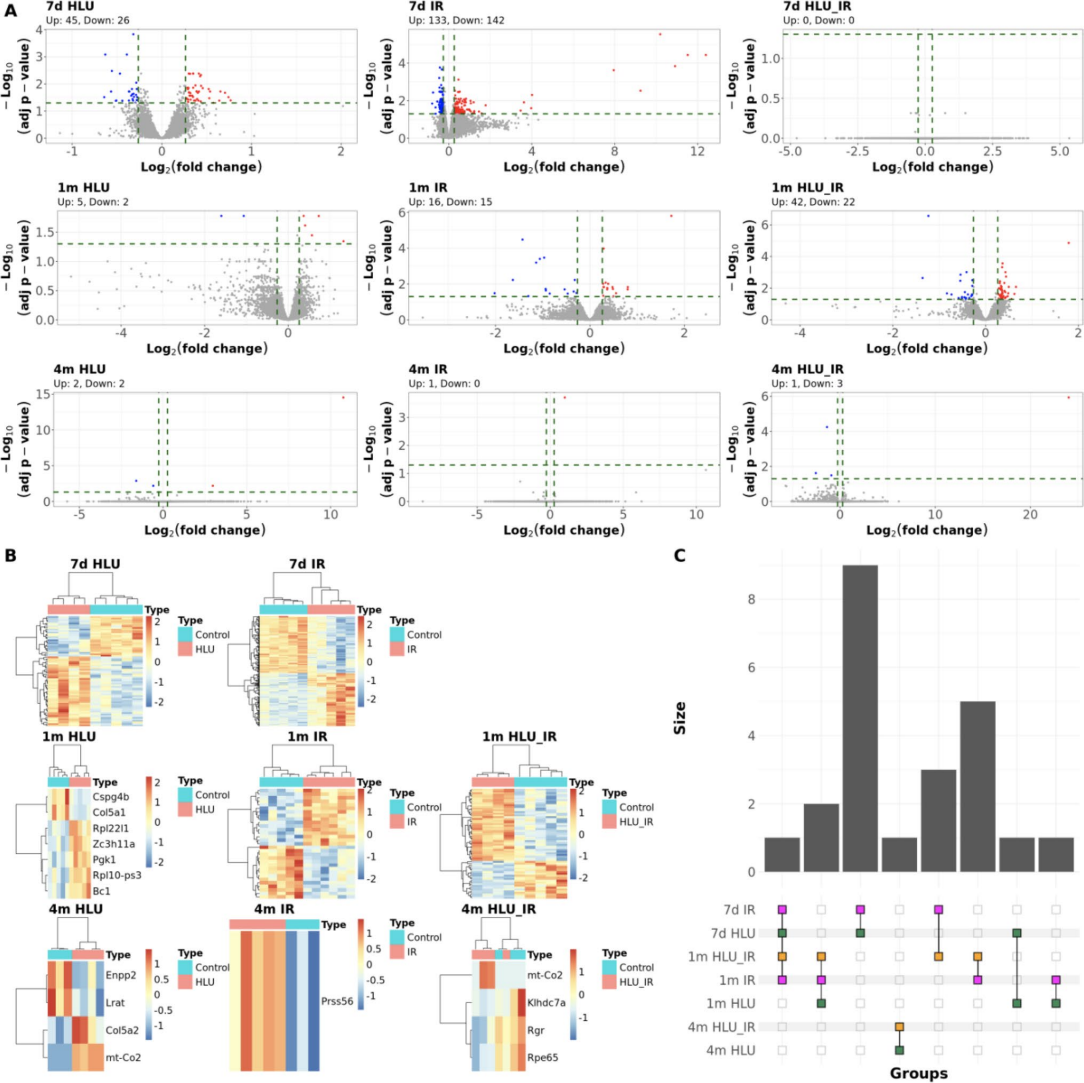
Differential gene expression in exposure groups. (**A**) Volcano plots showing DEG across each exposure condition (columns) at different time points (rows). Genes are colored by up-regulation (red) or down-regulation (blue) in exposed vs. control groups. Dashed green lines denote differential expression cutoffs of 0.05 and 0.263 for adjusted *p*-value and magnitude of log_2_(fold-change), respectively. Counts for up- and down-regulated genes in each exposure group are listed above each plot. **(B)** Heatmaps corresponding to the hierarchical clustering analysis of DEG in each group. Relative variance stabilizing transformed (VST) gene counts across samples are displayed as colors ranging from blue (low) to red (high) as shown in the key. Rows and columns are clustered using correlation distance and correspond to genes and samples, respectively. Exposure or control status are shown with colored bars at the top of each heatmap. **(C)** Upset plot showing DEG shared between at least two of the nine groups. The Y-axis represents the number of genes in a given intersection set. The X-axis lists different intersection sets and is ordered by the number of intersecting groups in a given set. Dots are colored by exposure condition (magenta for IR, green for HLU, orange for combination). *DESeq2* was used for differential gene expression analysis and R packages *ggplot2*, *pheatmap* and *ComplexUpset* were used for visualization. *DEG* differentially expressed genes, *HLU* hindlimb unloading, *IR* irradiation.

Post-exposure timepoints had different global expression patterns that were visible in principal component analysis where samples clustered more closely based on time point/age of mice instead of the exposure condition (Supplementary Fig. 1A). For both gene expression and methylation, each experimental group (Supplementary Table S1) was compared to a control group (no radiation or HLU) with matched post-exposure duration, and significant differential expression was identified in eight out of the nine comparisons (Table 1, Fig. 1A,B). While the samples clustered by time point/age based on global gene expression, they could be separated by exposure condition within each time point when genes were selected based on differential expression (Supplementary Fig. 1B, Fig. 1B).

**Table 1:** Number of differentially expressed genes and differentially methylated genes across exposure groups.

	7 days		1 month		4 months	
	DEG	DML/DMR	DEG	DML/DMR	DEG	DML/DMR
HLU	**71**	**743**	**7**	**716**	**4**	**134**
**↓**	26	173	2	67	2	55
**↑**	45	570	5	649	2	79
IR	**275**	**408**	**31**	**288**	**1**	**107**
**↓**	142	147	15	30	0	42
**↑**	133	261	16	258	1	65
HLU + IR	**0**	**374**	**64**	**957**	**4**	**451**
**↓**	0	78	22	170	3	154
**↑**	0	296	42	787	1	297

DEG (|log_2_(fold-change)|≥ 0.263 and adjusted *p*-value ≤ 0.05) and DMG (genes containing CpG loci or regions with |percent methylation difference|≥ 10 and adjusted *p*-value ≤ 0.05) are listed in bold for each experimental group compared to its matched control. DEG counts are separated by down (**↓**) or up (**↑**) regulation; DML/DMR counts are separated by hypo (**↓**) or hyper (**↑**) methylation. *DEG* differentially expressed genes, *DML* differentially methylated loci, *DMR* differentially methylated regions, *HLU* hindlimb unloading, *IR* irradiation.

The number of DEGs peaked at 7 days for groups exposed to only hindlimb unloading (HLU) or irradiation (IR) and showed a subsequent decline. The 275 DEG identified in 7-days IR were enriched in ocular disease terms (Supplementary Table S2) whereas the 71 DEG identified in HLU alone had no significant enrichment of disease pathways. In the 1-month IR group, 7 out of the 31 DEG showed > twofold change (down-regulated: *Pon1, Sult1c1, Ermn, Stra6, Tmem72, Gm24514;* up-regulated: *Bc1*). *Stra6* and *Pon1* are known to be involved in microphthalmia and diabetic retinopathy, respectively and *Bc1* affects vascular development^14^ and structural plasticity^15^. *Prss56*, a gene implicated in human and murine refractive development and myopia^16^, was the only DEG at 4 months after exposure to IR alone.

The highest number of DEG within HLU + IR (combination of hindlimb unloading and irradiation) was detected at 1 month post-exposure with no DEG at 7 days. The 64 DEG at 1 month included 20 genes with enrichment (adjusted *p*-value 8.24e-08) in the *somatodendritic compartment* of the neuron where synaptic inputs are received and integrated, and 15 genes known to be targets of *mmu-miR-466*, a miRNA that regulates age-related macular degeneration (AMD)^17^ (Supplementary Table S3).

#### Overlapping differentially expressed genes across exposure groups

Nine DEG were common between the HLU-only and IR-only groups at 7 days (down-regulated: *Arl2bp, Chrna6, Egln1, Map2k1, Stmn2*; up-regulated: *Ccn2, Mbd6, Sox9, St6galnac2, Zfp36l1*) (Fig. 1C). *Bc1* and *Pgk1* were significantly up-regulated in all exposure groups at 1 month. Zinc finger protein *Zc3h11a* was significantly down-regulated in the HLU only group at 7 days but up-regulated at 1 month. *Zc3h11a* expression has been shown to be altered in the murine retina and sclera in an experimental myopia mouse model^18^. Only a single common DEG was observed between the HLU + IR and HLU groups at 4 months (*mt-Co2*; up-regulated). *mt-Co2* is in the mitochondrial inner membrane and is an essential subunit of cytochrome c oxidase, the terminal component of the mitochondrial respiratory chain^19^. An integrated analysis of mammalian spaceflight data has reported the kidney to show an induction of *mt-Co2*^20^.

### Differential DNA methylation

**Figure 2:**
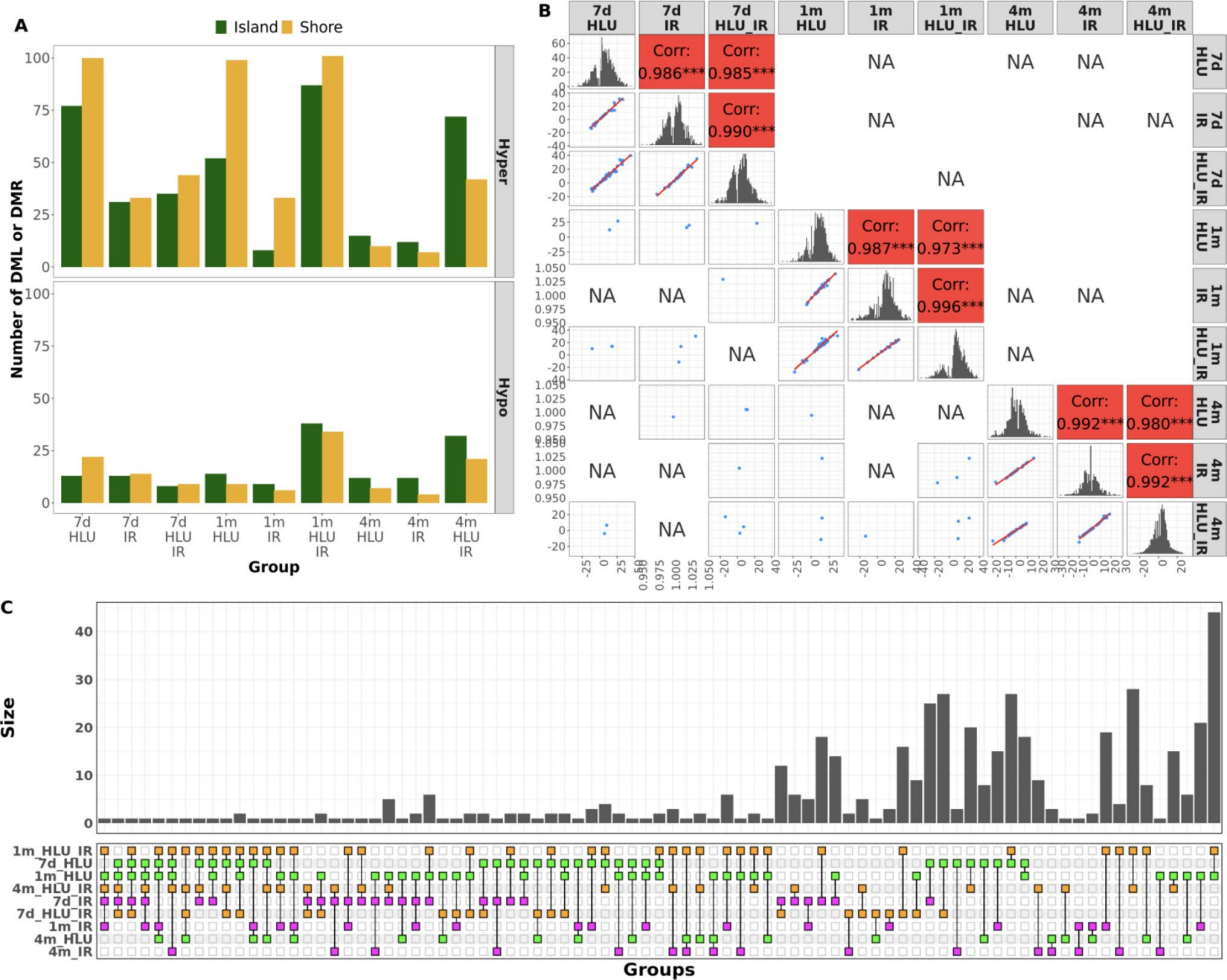
Differential methylation in exposure groups. (**A**) Total number of differentially methylated loci and regions within genes with hyper- or hypo-methylation in each exposure group categorized by whether they overlap with known CpG islands or shores. **(B)** Pairwise correlation plots for common differentially methylated sites. Differentially methylated loci (DML) with an adjusted *p*-value ≤ 0.05 were selected from each of the nine groups and pairwise correlation for methylation difference was plotted for any given pair. The diagonal shows distribution of methylation difference (%) between exposure and matched control group. Pearson correlation value and significance are displayed on the right of the diagonal, and scatter plots with methylation differences for common DML from any two given groups are displayed on the left. Only significant correlations (> 3 shared DML and *p*-value ≤ 0.05) are displayed. **(C)** Upset plot showing differentially methylated genes (DMG), defined as genes with their promoter or gene body containing a DML or DMR (adjusted *p*-value ≤ 0.05, |methylation difference|≥ 10%), shared between at least two of the nine groups. The Y-axis represents the number of genes in a given intersection set. The X-axis lists different intersection sets and is ordered by the number of intersecting groups in a given set. Dots are colored by exposure condition (magenta for IR, green for HLU, orange for combination). R package *MethylKit* was used for differential methylation analysis, *genomation* and *GenomicRanges* were used for annotation of DML, and *ggplot2*, *ggpairs* and *ComplexUpset* were used for visualization. *DML/R/G* differentially methylated loci/region/gene, *HLU* hindlimb unloading, *IR* irradiation.

The 1-month HLU + IR group showed the highest total number (957) of differentially methylated loci (DML) and regions (DMR) in known genes (Table 1, Fig. 2A). In all exposure groups, hypermethylation was more frequent than hypomethylation. Among the hypermethylated loci, a higher proportion of methylation differences were associated with CpG shores than with islands except in all three exposures at 4 months (Fig. 2A). Conversely, more hypomethylated loci overlapped with CpG islands than with shores in most of the groups except in all three groups at 7 days. The exposure groups at 4 months contained a higher proportion of total DML within CpG islands than shores compared to the other time points. Top 1% DML (roughly equivalent to methylation difference ≥ 20%; Supplementary Fig. 2) within CpG islands and exclusive to 4 months post-exposure were found in 17 genes across HLU (hypo: *Utp14a*; hyper: *Dlgap3*), IR (hypo: *Gm7855, Rtn4rl2*; hyper: *Maz, Shank3*), and HLU + IR (hypo: *Bag3*; hyper: *Aspdh, Map7d1, Map7d2, Morc4, Pcdh1, Pdzd11, Pik3ap1, Rtn4rl2, Suv39h1, Xk*).

#### Correlation in methylation differences across exposure groups

Pairwise analysis of all methylated loci in genes were compared across our 9 exposure groups (Fig. 2B). The highest correlation in methylation difference across different exposures was observed at the same post-exposure time point. Hypermethylation of *Bclaf3* promoter was observed in four groups (~ 15% in 1m HLU, 4m IR and 4m HLU + IR, and 26% in 1m HLU + IR), and another CpG island in *Tmc8* promoter was common across all three exposure groups at 7 days (10% hypermethylation). The top DMLs shared across all three exposures were in *Cav2* at 7 days (30% hypermethylation), *Grip2* at 1 month (20% hypermethylation), and *Sorbs2* at 4 months (8% hypermethylation).

#### Overlapping differentially methylated genes across exposure groups

A number of genes containing DML or DMR were observed in multiple exposure groups and post-exposure time points (Fig. 2C). *Bcl11b, Bclaf3, Gse1, Necab2, Plec* and *Tafa5* were each differentially methylated in five out of the nine groups. Six genes (*Adamts5, Gse1, Rbm15b, Fscn2, Satb1, Tenm3*) were shared across different time points after radiation exposure, with *Adamts5* and *Fscn2* promoters being hypermethylated even after 4 months of exposure to radiation alone. The promoter region of *Pcdh19,* a protocadherin, was hypermethylated (> 20% methylation difference) at all three time points for HLU + IR and also in HLU alone at 7 days. *Bclaf3* was hypermethylated in five groups (HLU at 7 days and 1 month, HLU + IR at 1 month and 4 months, and IR and 4 months), and CpG islands in *Hmgb3* promoter were hypermethylated in HLU and HLU + IR at both, 7 days and 1 month.

### Biological processes affected by low-dose radiation and hindlimb unloading across multiple conditions and post-exposure time points

#### Shared and exclusive biological processes across exposures and time points based on differential expression

**Figure 3:**
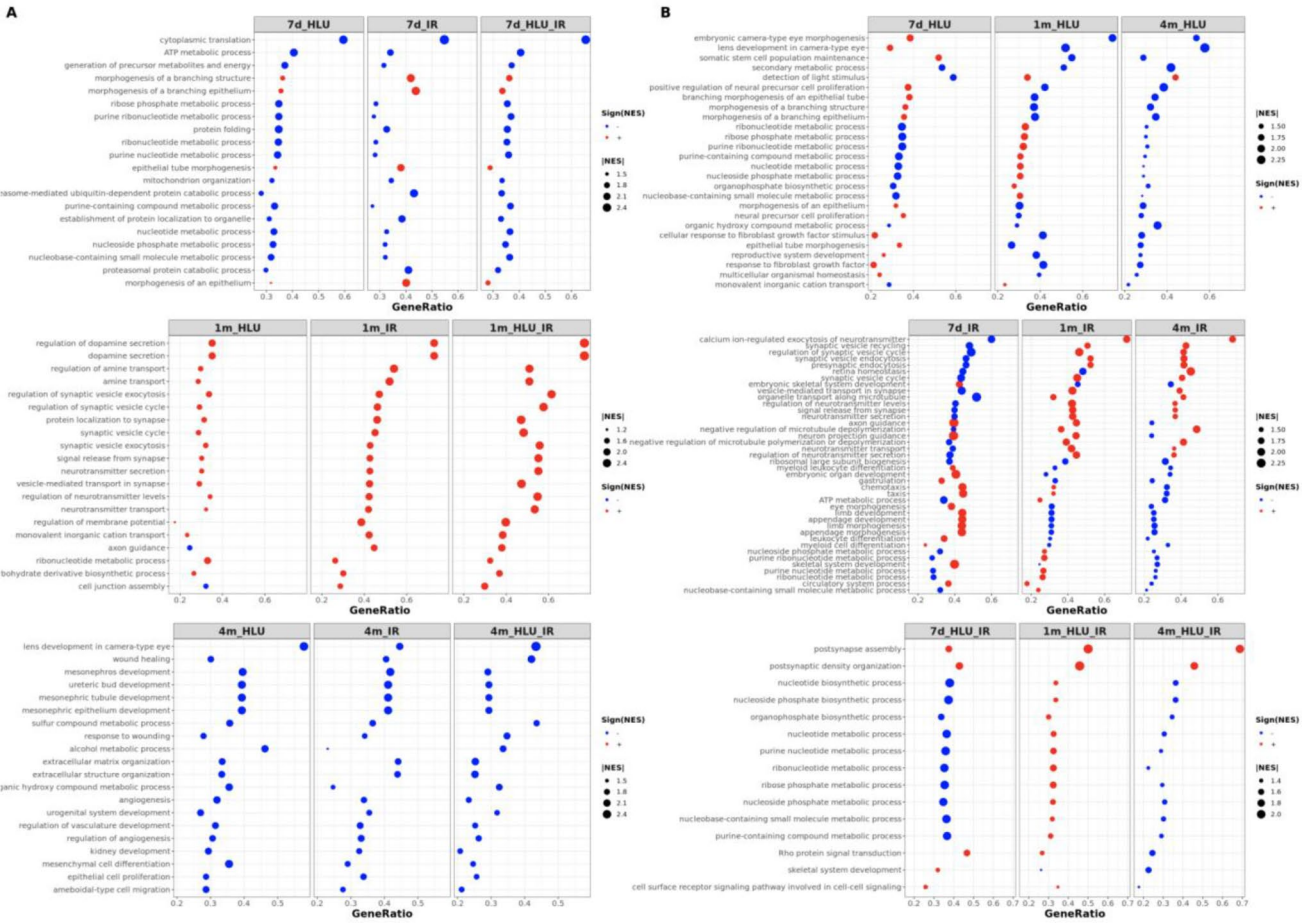
Common biological processes enriched in different exposure groups and at different time points. **(A)** Top 20 significantly enriched BP common across all exposure conditions for a given time point. **(B)** Significantly enriched BP common across all time points for a given exposure condition. Size of each point represents magnitude of normalized enrichment score (NES) while the color indicates activation (red) or suppression (blue) of the BP in the given exposure group compared to time point-matched controls. The Y-axis provides a description of common GO BP and the X-axis lists the ratio of genes that belong to a given gene set to the total number of genes in the gene set. *ClusterProfiler* was used for gene set enrichment analysis and the R package *ggplot2* was used for visualization. *BP* biological processes, *HLU* hindlimb unloading, *IR* irradiation, *NES* normalized enrichment score.

The top BP significantly enriched across all conditions 7 days after exposure included *morphogenesis of epithelium*, *ATP metabolism*, and *nucleotide metabolism* (Fig. 3A, Supplementary Table S4). Processes related to *epithelial morphogenesis* were activated in the exposure groups, whereas all other processes were suppressed across the three conditions. All shared processes were activated in the 1-month groups, with the exception of *axon guidance* and *cell junction assembly*, which were suppressed in the radiation only group. Common processes in the 1-month groups were related to *amine transport*, *regulation of membrane potential*, *synaptic vesicle exocytosis*, and *neurotransmitter transport*. At 4 months post-exposure, the top processes shared across the three exposure conditions were suppressed. These included *lens development in camera-type eye, wound healing, angiogenesis,* and *sulfur compound metabolic process*.

Processes that were significantly enriched at all post-exposure time points for a given exposure condition were evaluated (Fig. 3B, Supplementary Table S4). *Nucleotide metabolism* was suppressed at 7 days and 4 months but activated at 1 month in mice subjected to microgravity alone, whereas *epithelial tube morphogenesis* and *lens development* were activated at 7 days but suppressed at all later time points, and *detection of light stimulus* was suppressed at 7 days and subsequently activated at 1 month and 4 months. *Retina homeostasis* was suppressed in the radiation only groups at 7 days and 1 month but activated at 4 months, *eye morphogenesis* was activated at 7 days and suppressed subsequently, and *synaptic vesicle endocytosis* was suppressed at 7 days but activated at all later time points. In the combination groups, *postsynapse assembly* was activated at all time points, *nucleotide metabolism* was activated only at 1 month, and *Rho signal transduction* was activated at 7 days and 1 month followed by suppression at 4 months. *Nucleotide metabolism* and related processes were significantly enriched in all nine groups with consistent activation at 1 month and suppression at 7 days and 4 months. *Pdk1* was within the gene set for *nucleotide metabolism* processes across all nine groups, and its expression was decreased at 7 days and 4 months but increased at 1 month in all exposure groups (Supplementary Fig. 3). *Pdk1* is a kinase known to play a key role in regulation of glucose and fatty acid metabolism, and is involved in cellular response to hypoxia^21^.

As a complementary approach to examining shared processes, BP exclusive to each exposure condition (enriched in ≥ 2 post-exposure time points), or exclusive to each post-exposure time point were also examined (Table 2, Supplementary Table S5). Processes related to microtubule dynamics were enriched exclusively in HLU alone. IR-specific BP included *DNA conformation changes*, *Golgi organization*, *protein-DNA complex assembly*, and *type I interferon response*. The BP activated exclusively in IR but not enriched in HLU + IR at 7 days included *response to wounding, innate immune response, regulation of interleukin-6 production, heart morphogenesis, lipid metabolism,* and *epithelial cell proliferation*. The processes suppressed exclusively in IR were related to *telomere organization, DNA repair, ncRNA metabolism, detection of light stimulus,* and *organelle localization by membrane tethering*. Lastly, BP exclusive to HLU + IR were *postsynaptic density organization* and *regulation of AMPA receptors*. Each post-exposure time point was associated with distinct BP enriched across multiple conditions only at the given time point. At 7 days, BP related to *proteasomal protein catabolism* were suppressed in all groups, and *mammary gland morphogenesis* was activated in IR and HLU + IR. Oncogenesis in the retina and breast have been linked by the regulation of cyclin D1^22^; the gene encoding cyclin D1, *Ccnd1*, was significantly up-regulated 7 days after exposure to IR but was not differentially expressed at the later time points. *mRNA catabolic process* (suppressed) and *ion transmembrane transport* (activated) were among the BP exclusive to 1 month post-exposure. At 4 months, *glycerolipid metabolism*, *pigmentation*, and *brown fat cell differentiation* were suppressed.

**Table 2:** Representative biological processes enriched exclusively under each exposure condition or timepoint.

Category	GO ID	GO biological process	Adjusted *p*-value
**Exposure**			
HLU	GO:0099118	Microtubule-based protein transport	8.54E−04
	GO:0048872	Homeostasis of number of cells	1.49E−03
	GO:0005996	Monosaccharide metabolic process	3.03E−03
	GO:0030308	Negative regulation of cell growth	4.99E−03
	GO:1901653	Cellular response to peptide	9.58E−03
IR	GO:0000209	Protein polyubiquitination	1.20E−05
	GO:0042147	Retrograde transport, endosome to Golgi	4.33E−04
	GO:0065004	Protein-DNA complex assembly	1.02E−03
	GO:0002067	Glandular epithelial cell differentiation	2.77E−03
	GO:0034340	Response to type I interferon	9.65E−03
	GO:0098801	Regulation of renal system process	1.56E−02
HLU + IR	GO:0099084	Postsynaptic specialization organization	7.60E−05
	GO:2000311	Regulation of AMPA receptor activity	1.09E−04
	GO:0099565	Chemical synaptic transmission, postsynaptic	2.05E−04
**Timepoint**			
7d	GO:0030879	Mammary gland development	7.12E−07
	GO:0022406	Membrane docking	2.05E−06
	GO:0061136	Regulation of proteasomal protein catabolic process	5.82E−06
	GO:0090174	Organelle membrane fusion	1.42E−04
	GO:0070972	Protein localization to endoplasmic reticulum	6.69E−04
	GO:0045747	Positive regulation of Notch signaling pathway	1.10E−03
	GO:0044706	Multi-multicellular organism process	1.63E−02
	GO:0045576	Mast cell activation	2.07E−02
1m	GO:0042391	Regulation of membrane potential	2.72E−21
	GO:0034765	Regulation of ion transmembrane transport	6.64E−17
	GO:0007626	Locomotory behavior	3.25E−12
	GO:0050806	Positive regulation of synaptic transmission	1.09E−10
	GO:0050770	Regulation of axonogenesis	2.84E−09
	GO:0006402	mRNA catabolic process	9.05E−08
	GO:0019233	Sensory perception of pain	1.61E−07
	GO:0048588	Developmental cell growth	3.42E−06
	GO:0010770	Positive regulation of cell morphogenesis involved in differentiation	7.78E−06
	GO:0043087	Regulation of GTPase activity	7.00E−05
	GO:0043279	Response to alkaloid	1.77E−04
	GO:1901214	Regulation of neuron death	2.30E−04
	GO:0002244	Hematopoietic progenitor cell differentiation	3.36E−03
	GO:0008037	Cell recognition	7.00E−03
4m	GO:1905521	Regulation of macrophage migration	1.81E−05
	GO:0043473	Pigmentation	2.35E−05
	GO:0046486	Glycerolipid metabolic process	3.66E−05
	GO:0031669	Cellular response to nutrient levels	7.95E−05
	GO:0050873	Brown fat cell differentiation	8.04E−04
	GO:1902229	Regulation of intrinsic apoptotic signaling pathway in response to DNA damage	6.45E−03
	GO:0055076	Transition metal ion homeostasis	6.67E−03

*REVIGO* was used for reducing significant biological processes (adjusted *p*-value ≤ 0.05) exclusive to each exposure condition (HLU, IR, HLU + IR) or timepoint (7d, 1m, 4m) to representative non-redundant terms.

#### Over-represented biological processes based on differential methylation

Over-representation analysis (ORA) was applied separately to hypo- and hypermethylated genes, and only the hypermethylated genes resulted in significantly over-represented processes (Supplementary Table S6). ORA is conducted by iteratively counting the number of genes shared between a preselected input gene set (e.g., genes differentially methylated based on cutoffs for significance and magnitude of change) and each annotated gene set (e.g., all genes in a GO category), and applying a hypergeometric test to determine the statistical significance of the overlap^23^. Since all the groups showed differential methylation in a sizable set of genes compared to differential expression, ORA was suitable for methylation whereas gene set enrichment analysis (GSEA) was more appropriate for gene expression as it does not require an arbitrary cutoff for selecting significant genes. All genes overlapping with at least one covered CpG site were used as the gene background for ORA. Using a Benjamini–Hochberg adjusted *p*-value of 0.05 as the cutoff, nine processes were enriched in 4-month HLU + IR and included *regulation of Wnt signaling*, *neurogenesis, nervous system development, cell projection organization, cell surface receptor signaling, pattern specification process* and *morphogenesis of a polarized epithelium.* Genes with hypermethylation and involved in the regulation of these processes included oncogenes and tumor suppressors such as *Wnt3, Trp53, Trp73, Sox10, Rap1gap, Paqr3, Kit* and *Bex1.* The promoter region of the brain expressed X-linked gene, *Bex1*, contained four hypermethylated sites overlapping a CpG island. The gene has been reported to show promoter hypermethylation in malignant glioma cell line specimens^24^. Analysis based on KEGG pathways also showed an enrichment for *Wnt signaling* (raw and adjusted *p*-value of 0.005 and 0.3, respectively). Only one other group, 1-month HLU, showed over-representation for a biological process at an adjusted *p*-value cutoff of 0.05 (*actin filament-based process*).

### Integrated analysis of genes and biological processes impacted at transcriptomic and epigenomic levels

A total of 23 genes showed changes in expression and methylation at an adjusted *p*-value cutoff of 0.05 (Table 3). *Eef1a1*, a protein involved in peptide chain elongation, was down-regulated and hypomethylated in groups exposed to radiation or microgravity 7 days post-exposure. Crystallin *Crybb3* and signal-induced proliferation-associated *Sipa1l3,* both implicated in cataract, were up-regulated and hypermethylated 7 days after exposure to IR or HLU, respectively. Fibroblast growth factor 1 (*Fgfr1*) has been known to mediate photoreceptor rescue effect in response to retinal injury^25^, and the gene was up-regulated and hypomethylated 7 days after radiation. *Ncor2*, a gene in the Notch signaling pathway, was up-regulated with hypermethylation in the gene body in 7 days IR. Plectin *Plec*, a cytolinker protein involved in cytoskeletal organization, was up-regulated and hypermethylated. Filamin *Flnb* was also up-regulated and hypermethylated and is known to be involved in microvascular development^26^. No common differentially expressed and methylated genes were found in any of the exposure groups at 4 months.

**Table 3:** Genes with statistically significant expression and methylation changes.

Group	Gene	Log_2_fold-change (adj. *p*-value), % methylation difference (adj. *p*-value)	Differential methylation genomic context (gene body vs. promoter)
7d HLU	*Cdk14* (cyclin dependant kinase)	− 0.24 (0.01), 2.81 (0.04)	Promoter
	*Eef1a1* (eukaryotic translation elongation factor)	− 0.23 (0.02), − 11.74 (0.03)	Promoter
	*Fam222a* (aggregatin)	0.30 (0.004), − 6.76 (0.03)	Gene body
	*Pitpnm3* (membrane-associated phosphatidylinositol transfer domain-containing)	0.20 (0.01), 9.90 (0.02)	Promoter
	*Sipa1l3* (signal induced proliferation associated)	0.20 (0.02), 15.44 (0.009)	Gene body
	*Sox9* (SRY-box transcription factor)	0.35 (0.004), − 5.74 (0.03)	Gene body
7d IR	*BC031181* (cDNA sequence)	− 0.33 (0.04), 26.70 (0.03)	Promoter
	*Crybb3* (crystallin)	3.47 (0.03), 16.40 (0.006)	Promoter
	*Dcp1b* (decapping mRNA)	0.32 (0.03), − 11.22 (0.04)	Gene body
	*Eef1a1* (eukaryotic translation elongation factor)	− 0.25 (0.002), − 13.72 (0.0002)	Promoter
	*Fgfr1* (fibroblast growth factor receptor)	0.61 (0.02), − 0.65 (0.02)	Promoter
	*Flnb* (filamin)	0.38 (0.03), 11.11 (0.04)	Gene body
	*Mbd6* (methyl-CpG binding domain)	0.31 (0.005), 8.96 (0.002)	Promoter
	*Ncor2* (nuclear receptor corepressor)	0.29 (0.02), 20.20 (0.0003)	Gene body
	*Plec* (plectin)	0.36 (0.02), 11.95 (0.0008)	Gene body
	*Ppm1a* (protein phosphatase)	− 0.22 (0.02), 0.79 (0.04)	Promoter
1m HLU + IR	*B4galt6* (membrane-bound glycoprotein)	0.36 (0.0004), − 3.89 (0.02)	Promoter
	*Kcnip3 (*potassium voltage-gated channel interacting)	0.35 (0.02), − 14.28 (0.04)	Promoter
	*Lamp5* (lysosomal associated membrane protein)	0.30 (0.03), 10.48 (0.03)	Promoter
	*Nacad* (NAC alpha domain containing)	0.31 (0.003), 20.22 (0.03)	Gene body
	*Rundc3a (*RUN domain containing)	0.29 (0.01), 23.99 (0.04)	Promoter
	*Sphkap (*SPHK1 interactor, AKAP domain containing)	0.22 (0.02), 17.98 (0.04)	Promoter
	*Tle3* (transcriptional co-repressor)	0.36 (0.0002), 7.30 (0.05)	Gene body

Genes that are differentially expressed in a given group (adjusted *p*-value ≤ 0.05) and also contain at least one differentially methylated locus or region (adjusted *p*-value ≤ 0.05) are listed. Log_2_(fold-change) for expression difference, percentage methylation difference, and location of DML or DMR within gene context (promoter vs. gene body) are also included. Adjusted *p*-values for expression and methylation differences are also provided. *HLU* hindlimb unloading, *IR* irradiation, *DML* differentially methylated locus, *DMR* differentially methylated region.

The biological processes and constituent genes shared within a given group with over-representation based on methylation data and enrichment in expression analysis were also obtained (Table 4, Supplementary Table S7). The highest number of overlapping processes were observed in 4-months HLU + IR, with *Wnt signaling pathway* being one of the shared BP. *Actin filament-based process* was enriched in 1-month HLU and 4-months HLU + IR based on both, differential expression and methylation. Processes related to *ion transmembrane transport* were activated in the 1-month HLU + IR group. Shared genes included ephrin *Ephb2*, which is also dysregulated in the murine brain following hindlimb unloading^6^. Calcium binding protein *Cabp1* and calcium voltage-gated channel subunits *Cacna1c* and *Cacna1h* were among the genes involved in ion transport; another calcium voltage-gated channel subunit, *Cacna2d4*, was also impacted in the murine retina after spaceflight^10^.

**Table 4:** GO biological processes and related genes common between RNA-Seq and methylation.

Group (Total no. of BP)	GO Biological Process	Overlapping genes
7d_HLU (9)	Multicellular organismal homeostasis	*Alox12b, Crtc1, Edn2, Epas1, Igf1r, Mbp, P2rx7, Stat3, Tmem119, Trim32*
	Trans-synaptic signaling	*Adcyap1, Atp2b2, Cacnb4, Chat, Itgb1, Itpka, Lrfn2, Rapgef2*
	Positive regulation of cold-induced thermogenesis	*Epas1, Igf1r*
	Anterograde trans-synaptic signaling	*Adcyap1, Atp2b2, Cacnb4, Chat, Itgb1, Itpka, Lrfn2, Rapgef2*
	Chemical synaptic transmission	*Adcyap1, Atp2b2, Cacnb4, Chat, Itgb1, Itpka, Lrfn2, Rapgef2*
	Cell junction assembly	*Fgf13, Nrxn1, Sdk2*
	Synaptic signaling	*Adcyap1, Atp2b2, Cabp1, Cacnb4, Chat, Itgb1, Itpka, Lrfn2, Rapgef2*
	Regulation of neurotransmitter levels	*Atp1a2, Cacnb4, Chat, Itgb1, Pebp1*
	Temperature homeostasis	*Edn2, Epas1, Igf1r, Stat3*
7d_IR (17)	Cell morphogenesis involved in differentiation	*Cdh23, Cul7, Ephb2, Fgfr3, Flnb, Kif1a, Lamc1, Plxna1, Ptprs, Rreb1*
	Myotube differentiation	*Adamts15, Adamts5, Plec*
	Forebrain development	*Ephb2, Fgfr3, Kif1a, Ncor2, Ptprs*
	Cellular component morphogenesis	*Cul7, Dab2ip, Ephb2, Fgfr3, Kif1a, Plec, Plxna1, Ptprs, Rreb1*
	Cell part morphogenesis	*Cul7, Dab2ip, Ephb2, Fgfr3, Kif1a, Plec, Plxna1, Ptprs, Rreb1*
	Plasma membrane bounded cell projection morphogenesis	*Cul7, Dab2ip, Ephb2, Fgfr3, Kif1a, Plxna1, Ptprs, Rreb1*
	Cell projection morphogenesis	*Cul7, Dab2ip, Ephb2, Fgfr3, Kif1a, Plxna1, Ptprs, Rreb1*
	Myoblast fusion	*Adamts15, Adamts5*
	Telencephalon development	*Ephb2, Ncor2, Ptprs*
	Cell–cell fusion	*Adamts15, Adamts5, Sh3pxd2a*
1m_HLU (8)	Negative regulation of cell differentiation	*Hmgb3, Mbnl3, Nfatc2, Nr1d1, Prdm16, Zfpm1*
	Actin cytoskeleton organization	*Ablim2, Cd2ap, Csrp2, Fhod3, Fmnl3, Foxj1, Frmd6, Iqgap3, Myo15, Myo18a, Myo1h, Pacsin1, Pakap, Plec, Rapgef3, Rhobtb1*
	Actin filament-based process	*Ablim2, Atp1a1, Cacna1d, Cd2ap, Csrp2, Fhod3, Fmnl3, Foxj1, Frmd6, Iqgap3, Myo15, Myo18a, Myo1h, Pacsin1, Pakap, Pde4d, Plec, Rapgef3, Rhobtb1*
	Actomyosin structure organization	*Csrp2, Fhod3, Frmd6, Myo18a, Plec, Rapgef3*
	Regulation of cell shape	*Fmnl3, Myo10, Pakap, Rhobtb1*
	Regulation of cell morphogenesis	*Bcl9l, Cpne5, Fmnl3, Myo10, Pakap, Rhobtb1, Rreb1*
	Regulation of glycolytic process	*Esrrb, Zbtb20*
	Regulation of nucleotide metabolic process	*Hpca, Prkag2*
1m_HLU_IR (5)	Cation transmembrane transport	*Akt1, Atp13a2, Cabp1, Cacna1c, Cacna1h, Cemip, Ephb2, Hcn3, Kcnc2, Kcnh1, Kcnn2, Nalf2, Nrxn1*
	Inorganic cation transmembrane transport	*Akt1, Cabp1, Cacna1c, Cacna1h, Cemip, Hcn3, Kcnc2, Kcnh1, Kcnn2, Nalf2*
	Regulation of ion transmembrane transport	*Akt1, Cabp1, Cacna1c, Cacna1h, Cemip, Ephb2, Hcn3, Kcnc2, Kcnh1, Kcnn2, Nrxn1*
	Cellular protein complex disassembly	*Apc2, Dmtn, Eml4, Nrg1, Nsf, Synj1*
	Cellular component disassembly	*Apc2, Atg2a, Dmtn, Eml4, Nrg1, Nsf, Ston1, Synj1, Vps13c*
4m_HLU_IR (52)	Actin filament-based process	*Cdh1, Lmod1, Plekhg2*
	Cellular response to growth factor stimulus	*Fgf5, Ntrk2, Prdm16*
	Morphogenesis of an epithelium	*Cdh1, Col5a1, Lama5, Notch2, Sox10, Wnt7b*
	Wnt signaling pathway	*Cdh1, Ptpru, Wnt7b*
	Collagen fibril organization	*Col5a1, Comp, Loxl1*
	Sensory organ morphogenesis	*Col5a1, Ntrk2*
	Cartilage development	*Comp, Wnt7b*
	Protein localization to plasma membrane	*Cdh1, Flot2, Lama5*
	Neuron projection guidance	*Lama5, Wnt7b*

Top ten BP (or lower if there are < 10 processes) enriched in a given group based on both, differential expression and differential methylation, are listed. The total number of BP for each group, and constituent genes that overlap for a given BP are also included. Full list of BP, constituent genes, and associated methylation difference and fold change values are included in Supplementary Table S7. *BP* biological process, *HLU* hindlimb unloading, *IR* irradiation.

## Discussion

Spaceflight is an environment to which Earth-based biological systems have not yet adapted. Changes in gravity as well as radiation exposure that differs from terrestrial radiation both in terms of intensity and type, are the two primary naturally occurring hazards the human body contends with during space travel. It is crucial to characterize the impacts of these hazards to ensure the crew and NASA as an agency are appropriately informed. While hazards will be experienced concurrently in spaceflight, important information about the potential interactions between hazards can reveal how impacts can be appropriately mitigated. Different countermeasure strategies will need to be employed if hazards modulate similar or divergent processes.

It is important to note the current study leverages data from a previously performed animal study where mice were exposed to gamma-rays and microgravity as analogs of space radiation and microgravity respectively. However, even with the inherent limitations of using analogs, the observed results still provide insights on processes related to retinal dysfunction due to individual and combined exposures, at different time points, allowing us to gain a preliminary understanding of longitudinal changes. Compared to expression results from other tissue types collected in accompanying studies, spleen data from 7 days post-exposure showed highest number of DEG in HLU alone^12^, brain data from 4 months had most DEGs in HLU + IR^6^, whereas the retina results presented here showed DEGs peaked in IR alone at 7 days, and HLU + IR at 1 month. Combined stressors can induce complex responses further regulated by factors such as radiation type, post-exposure duration, and tissue type. Given the conflicting results from various studies addressing synergies between microgravity and radiation and their impact on DNA damage repair and cellular repair^27^, further work is needed to understand their interplay.

Biological processes shared across, or exclusive to, exposure conditions and post-exposure stages were identified (Fig. 1, Table 2, Supplementary Table S5), and *nucleotide metabolism* was enriched in all the groups with activation at 1 month, suppression at 7 days and even lower at 4 months for all exposures. The suppression of nucleotide metabolism has been shown to play a role in the establishment and maintenance of the stable growth arrest of oncogene-induced senescence^28^. Further work is required to understand the mechanism behind the activation at 1 month with a return to being suppressed at 4 months, and the specific role of *Pdk1* in process modulation.

Several genes known to be implicated in ocular diseases were found to be impacted at the transcriptomic and epigenomic levels. A subset of the 7-days radiation only DEG (*Cryaa, Cryba1, Cryba2, Cryba4, Crybb1, Crybb2, Crybb3, Crygs, Bsfp1*) were enriched in *nuclear and congenital cataracts*, however, these genes were not dysregulated at 1 month or 4 months. A key component of the MAP kinase signal transduction pathway, *Map2k1*, was down-regulated in the IR and HLU + IR groups at 7 days but up-regulated at 1 month; MAPK signaling has been shown to be involved in AMD pathogenesis^29^. *Bc1*, a non-coding RNA known to affect vascular development^14^ and structural plasticity^15^ was significantly up-regulated in all three exposures at 1 month. Four months after radiation, retention of hypermethylation was observed in genes involved in eye development and pathogenesis including *Myc*-associated zinc-finger transcription factor *Maz*^30^*,* disintegrin *Adamts5* involved in AMD*,* and *Fscn2* with a role in photoreceptor disk morphogenesis. Interestingly, *Adamts5* was previously observed to be dysregulated in the murine retina after spaceflight^11^*.* Additionally, early differential methylation (only at 7 days and 1 month) was observed in *Hmgb3,* a member of the high-mobility group superfamily and a transcription factor known to be impacted by spaceflight^10^.

A total of 23 genes shared differential expression and methylation (Table 3). Of these, 14 had differential methylation in the promoter region and 9 in the gene body with negative correlation between expression and methylation changes in 3 and 6 genes, respectively. The rest showed a positive correlation. Recent studies have expanded the understanding of effects of DNA methylation on gene expression, especially in cancers^31^*.* Along with the traditional methylation-induced gene silencing, there have been patterns of consistently positive or negative correlations for all CpG sites associated with specific genes. More work is needed to understand the variation in the impact of DNA methylation on expression across different tissue and cell types, genes, and the location of CpG sites. High-mobility group containing *Sox9* is known to be involved in retinogenesis^32^ and was up-regulated in 7-days HLU and IR groups, and hypomethylated in 7-days HLU. *Ppm1a* has been shown to play a crucial role in the wound healing-inflammation-angiogenesis axis in mice^33^, and was down-regulated and hypermethylated 7 days after radiation but not later. *Sphk1* plays a key role in the regulation of inflammatory responses^34^; *Sphkap*, a modulator of *Sphk1*, was up-regulated and hypermethylated in 1-month HLU + IR. The glycoprotein *B4galt6* regulates astrocyte activation during CNS inflammation^35^ and was up-regulated and hypomethylated*.* Interestingly, *Sphk1* and glycoproteins *B4galt2* and *B4galt3* were also up-regulated in 7 days HLU + IR based on spleen data from the same set of mice^12^. Transcriptional co-repressor *Tle3*, involved in Wnt and Notch signaling pathways, was up-regulated and hypermethylated in 1-month HLU + IR.

Biological processes related to *Wnt signaling pathway* were significantly enriched in multiple groups based on differential expression, with activation in IR and HLU + IR at 7 days, and suppression in 1-month HLU and all three exposures at 4 months. *Wnt signaling pathway* is critically involved in cell–cell communication and regulates tissue homeostasis^36,37^. Altered activities may promote tissue degeneration^38^. Wnt signaling plays a role in eye organogenesis^32^ and genes in the pathway have been reported to be impacted by spaceflight^11^. The pathway has also been shown to be affected by spaceflight in 3D-cultured neural stem cells^39^ and human cardiovascular progenitor cells^40^. *Bclaf3* has been proposed to regulate proliferation/apoptosis by suppressing Wnt signaling in the mouse gastric epithelium^41^, and showed hypermethylation in the promoter within four of the groups including IR and HLU + IR at 4 months. Interestingly, genes involved in Wnt signaling also appeared as members in processes enriched in 4-months HLU + IR based on methylation data (*Wnt3, Wnt11, Wnt7b, Lrp6, Tnik;* Supplementary Table S6).

Among the BP shared between differential methylation and expression (Table 4), pathways related to *Wnt signaling* were also over-represented in 4-months HLU + IR. *Collagen fibril organization* was over-represented with three overlapping constituent genes: *Col5a1* encodes an alpha chain of type V collagen and the gene family forms a major component of the basement membrane of the corneal endothelium and related genes were altered in the mouse retina after spaceflight^11^, lysyl-oxidase like *Loxl1* has been implicated in pseudoexfoliation syndrome which is a major cause of glaucoma and cardiovascular complications^42^, and *Comp* plays a role in vascular wall remodeling^43^. *Actin filament-based process* was enriched in 1-month HLU and 4-months HLU + IR. Some of the gene members based on differential methylation in 1-month HLU are also known to be involved in *signaling by Rho GTPases (Rhoc, Rhobtb1, Arhgef10l, Cyfip2, Fmnl3, Fgd1, Iqgap3;* Supplementary Table S6)*.* Rho GTPases are key players in adaptation to microgravity and actin cytoskeleton dynamics in mammalian cells^44,45^. *Temperature homeostasis* was enriched in 7-days HLU, and has been shown to be impacted by hindlimb suspension in a previous study where the authors noted that unloading prevented the mice from curling up at night to regulate body temperature^46^. This difference in temperature regulation between the control and experimental groups should be considered while designing unloading experiments and interpreting the results.

Interestingly, the NASA Twins Study reported *Notch3* to show epigenetic discordance based on spaceflight blood samples^13^ and the gene was significantly up-regulated (twofold change) at 7 days post-irradiation in the mouse retina. *Rbx1* and *Ncor2*, also in the Notch signaling pathway, showed 33% hypermethylation in 4-months HLU + IR, and up-regulation and hypermethylation 7 days post-irradiation, respectively. The NASA Twins Study also reports a decrease during flight in *leucine rich alpha-2-glycoprotein 1*, a protein involved in retinal vascular pathology. *Lrg1* was down-regulated by more than fourfold (unadjusted *p*-value = 0.006) in 1-month HLU and significantly hypermethylated (16.8% methylation difference) in 4-months HLU + IR.

This multi-omics murine analysis highlights changes in gene expression, DNA methylation, and regulation of biological processes exclusive to, or shared between, exposure groups at different timepoints. Furthermore, there were expressed genes and processes that only presented themselves at the 4-month time point, which may be indicative of a longer post-exposure duration to understand the effects of low-dose radiation and microgravity on the retina. Wnt signaling has been shown to be perturbed in the murine retina after spaceflight^11^ and our results show discordance in the pathway in the 4-months HLU + IR group based on changes in expression and methylation. *Notch3* and *Lrg1* showed discordance during flight in The NASA Twins Study blood samples^13^ and also exhibit differential patterns in the murine retina in the current study. Hence, despite limitations mentioned below, our study provides confidence that murine models can provide an understanding of molecular mechanisms and pathways contributing to ocular function as a response to analogs for spaceflight stressors.

Our study comes with limitations. First, the number of samples was limited with only a total of 59 mice. Therefore, each exposure group at each time point was limited to 3–6 mice, thus limiting power. A follow-up study with more animals should be conducted to confirm these observations. Second, the animals were radiated by low-dose gamma rays which do not represent space radiation. Ground-based low-dose gamma or x-ray radiation studies are often used to study the effect of space-relevant radiation exposures (in terms of total dose) on different tissues/systems^6,47,48^ as to inform future space radiation experiments and form an initial understanding of the impacts across radiation doses and types. However, further studies should be implemented with higher dose gamma radiation and GCR simulations to leverage a more realistic space radiation analog. Third, a key limitation of applying bulk RNA-Seq and RRBS to a complex tissue such as the retina with ~ 140 cell types^49^ is that the approach averages signals over heterogeneous cell types at different transcriptomic states. As a result, true perturbations in, or correlation between, expression and methylation in a small cell population can be obscured by aggregated profiles. Fourth, caution is needed while comparing results between this study and The NASA Twins Study as biospecimen type and analysis time points differ between studies, and correlating time points across species with such different lifespans is challenging. Therefore, it is important to note that this analysis with its limitations only provides a preliminary signal for further investigation into understanding the longitudinal effect of spacelight hazard analogs on retina.

Overall, the results of this study support the development of a more robust understanding of the longitudinal molecular mechanisms implicated in the murine retina response to low-dose gamma irradiation and simulated microgravity. Further investigation into the interplay between these mechanisms and validation of results in multiple models may provide insights into key pathways with translational potential across biological systems and exposure types as well as targets for mitigation strategies.

## Methods

### Experimental conditions

The original study was conducted at Loma Linda University following recommendations in the Guide for the Care and Use of Laboratory Animals^50^ and was approved on April 30, 2014, by the Institutional Animal Care and Use Committee (IACUC) at Loma Linda University (Protocol number 8130028). A detailed description of the experimental setup has been published previously^51^. Briefly, a total of 59 six-month-old, female *C57BL/6 J* mice were housed in cages of same dimensions and subjected to one of four experimental conditions: sham control with no radiation or unloading (N = 15), hindlimb unloading (N = 14), low-dose irradiation (N = 15), and a combination of hindlimb unloading and low-dose irradiation (N = 15) for 21 days (Supplementary Table 1). Hindlimb suspension was used to model the unloading, fluid shift, and physiological stress aspects of the microgravity component. Gamma radiation was delivered using ^57^Co plates placed underneath the cages and a dose rate of 0.01 cGy/h was used to deliver a total dose of 0.04 Gy over a period of 21 days to simulate a space radiation environment. For the combination group, mice were exposed to unloading and simultaneously irradiated by ^57^Co plate placed underneath the cages. Mice from each exposure condition were then allowed a post-exposure period of 7 days, 1 month, or 4 months, following which mice were euthanized and retinas were collected and frozen. RNA and DNA libraries were constructed and used for RNA-Seq and RRBS. The resulting data was deposited to GeneLab where RNA-seq output was processed per NASA GeneLab RNA-seq pipeline^52^, and RRBS results are available in the form of raw sequencing reads. Unnormalized RNA-seq counts and raw RRBS FastQ files were downloaded from GeneLab and used for the current analysis.

### Differential gene expression analysis

Unnormalized RNA-seq counts were downloaded from GeneLab. Out of a total of 59 samples (Supplementary Table S1), three were excluded from expression analysis due to low RNA integrity number values or high ribosomal RNA contamination. These counts were generated with GeneLab RNA-seq consensus pipeline, which includes quality control, read trimming, mapping, and gene quantification steps. Full details of the pipeline including the parameters used for each individual tool have been previously published^52^. RNA-seq counts were subsequently analyzed with *DESeq2* (v1.30.1)^53^ where counts were first normalized using the median-of-ratios method followed by differential gene expression analysis comparing each experimental group to the corresponding post-exposure time point-matched control group. Differentially expressed genes were determined using cutoffs of 0.263 and 0.05 for log_2_(fold-change) and Benjamini–Hochberg adjusted *p*-value, respectively, to be consistent with analyses of brain and spleen tissues from the same set of mice^6,12^. R packages *ggplot2*^54^, *ComplexHeatmap*^55^ and *pheatmap*^56^ were used for visualization and generating heatmaps.

### Differential methylation analysis

Raw RRBS reads were downloaded from GeneLab and processed with nf-core/methylseq pipeline (https://github.com/nf-core/methylseq; v1.6.1 accessed on June 23^rd^, 2021). In short, *FastQC* (http://www.bioinformatics.babraham.ac.uk/projects/fastqc) was used for running quality checks on reads, adapter sequences were trimmed using *Trim Galore! for Cutadapt*^57^ without the *–rrbs* option as recommended for NuGEN RRBS Ovation kit, NuGEN’s diversity trimming script was run, and *Bismark*^58^ was used for alignment and methylation call extraction. Deduplication based on overlapping coordinates was skipped as it is not recommended for RRBS^59^. Two samples were excluded from methylation analysis due to GC content bias. Mean Phred quality score across each base position in the read was confirmed to be ≥ 20 (Supplementary Fig. 4), and *MethylKit* (v1.16.1)^60^ was used for detection of differentially methylated loci with a cutoff of 0.05 and 10% for a sliding linear model adjusted *p*-value^61^ and magnitude of methylation difference, respectively^11^. As a complementary approach, methylation differences were also calculated over 100-bp tiling windows to obtain differentially methylated regions^60^. Sites and regions were mapped to known genes (mm10 assembly) and known CpG islands using *genomation*^62^ and *GenomicRanges*^63^ packages. Promoter regions were defined as 2 kbp upstream and downstream of annotated transcription start sites. Differentially methylated genes were defined as genes containing at least one DML or DMR within their promoter region or gene body. For determination of overlap between differential expression and methylation, no thresholds for fold-change or methylation difference were used to allow for genes with modest, but still significant (adjusted *p*-value ≤ 0.05), changes to be observed.

### Gene set enrichment and over-representation analyses

GSEA for gene expression and over-representation analysis for methylation were conducted with the *ClusterProfiler* (v3.18,1) package^64^ against the biological process GO category using *gseGO* and *enrichGO* functions, and against KEGG pathways using *enrichKEGG*. Wald test statistics from *DESeq2* differential gene expression analysis were used to rank genes for enrichment analysis. Appropriate background gene lists were created by selecting all genes expressed in the retina or covered by at least one CpG site (within ± 2 kb of the gene) for expression (GSEA) and methylation (ORA) data, respectively. A Benjamini–Hochberg adjusted *p*-value cutoff of 0.05 was used for selecting enriched terms. *REVIGO*^65^ was used for reducing significant biological processes to a representative non-redundant set of terms. ORA was performed separately on hypo and hypermethylated genes to obtain significantly enriched GO biological processes (adjusted *p*-value ≤ 0.05) based on differential methylation in each group. Following adjustments were made to GSEA and ORA to allow for the discovery of overlapping candidate processes and genes between expression and methylation that can be further validated: (a) differentially methylated genes were defined as genes with their promoter or gene body containing a DML (adjusted *p*-value ≤ 0.05, |methylation difference|≥ 10%) or DMR (adjusted *p*-value ≤ 0.05, |methylation difference|≥ 2%); regulated methylation targets are routinely clustered into short genomic regions^66^ and a lower threshold for methylation difference in DMR (2%) is recommended for enrichment analysis since perturbed processes can be a cumulative effect of multiple DMR with small effects^67^, and (b) an adjusted *p*-value cutoff of 0.25 was used for detecting overlapping biological processes based on differential methylation and expression; GSEA guidelines recommend a 25% false discovery rate (FDR) for exploratory analyses as a more stringent threshold can exclude significant results even before they can be validated^68^. *Enrichr*^69^ and *ToppGene*^70^ were used for disease term and functional enrichment analysis.

## Supplementary Information

Supplementary Information 1.

Supplementary Information 2.

## Data Availability

Transcription profiling and methylation data were downloaded from NASA’s GeneLab platform (https://genelab-data.ndc.nasa.gov/genelab/accession/GLDS-203/; version 6). All software packages are open source and have been cited in the Methods section.
