# Supplementary material for: A multi-omics longitudinal study of the murine retinal response to chronic low-dose irradiation and simulated microgravity
Source: Sci Rep. 2022 Oct 7;12:16825. doi: 10.1038/s41598-022-19360-9 (PMC9547011; doi:10.1038/s41598-022-19360-9)
Supplement: Supplementary file 1 — Supplementary Information 1. [file 41598_2022_19360_MOESM1_ESM.docx]

# **A multi-omics longitudinal study of the murine retinal response to chronic low-dose irradiation and simulated microgravity**

Prachi Kothiyal^1^, Greg Eley^2^, Hari Ilangovan^3^, Katherine A. Hoadley^4^, S. Robin Elgart^5^, Xiao W. Mao^6^, Parastou Eslami^7^

^1^ SymbioSeq LLC, Ashburn, VA 20148, USA

^2^ Scimentis LLC, Statham, GA 30666, USA

^3^ Science Applications International Corporation (SAIC), Reston, VA 20190, USA

^4^ Department of Genetics, Lineberger Comprehensive Cancer Center, University of North Carolina at Chapel Hill, Chapel Hill, NC 27599, USA

^5^ University of Houston, Houston, TX 77204, USA

^6^ Basic Sciences, Loma Linda University, Loma Linda, CA 92350, USA

^7^ Universal Artificial Intelligence Inc., Boston, MA 02130, USA

## **Supplementary Data**

**Supplementary Figures**

**1A**


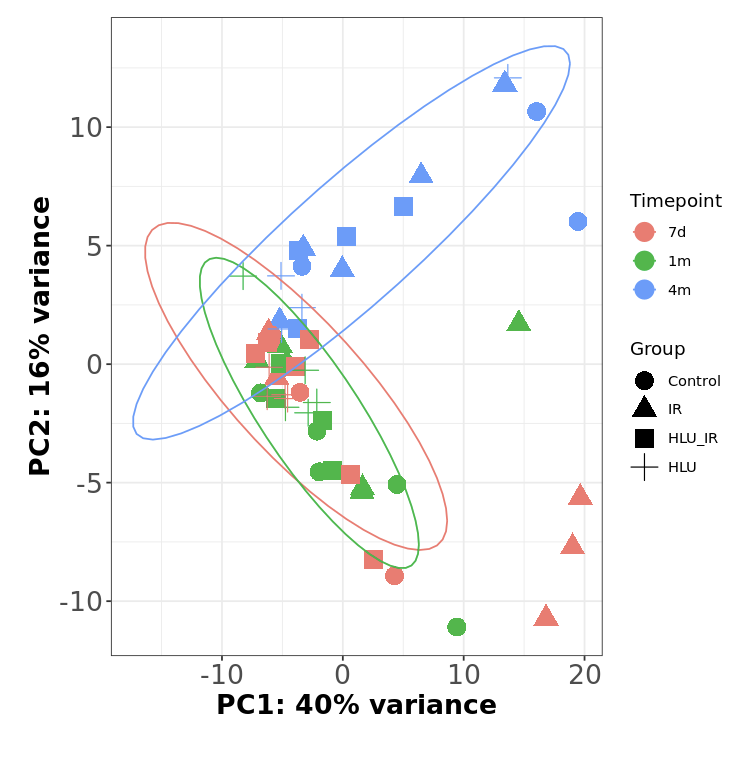


**1B**


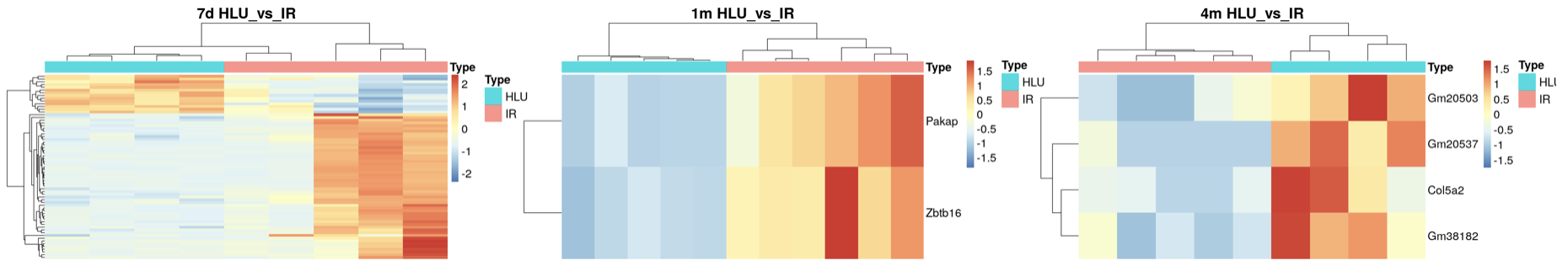


**Supplementary Figure 1:** **Clustering based on global expression data and differentially expressed genes. (A)** Principal component analysis using global gene expression data shows clustering by timepoint/age. **(B)** Hierarchical clustering using only differentially expressed genes shows separation between HLU and IR groups within each timepoint.


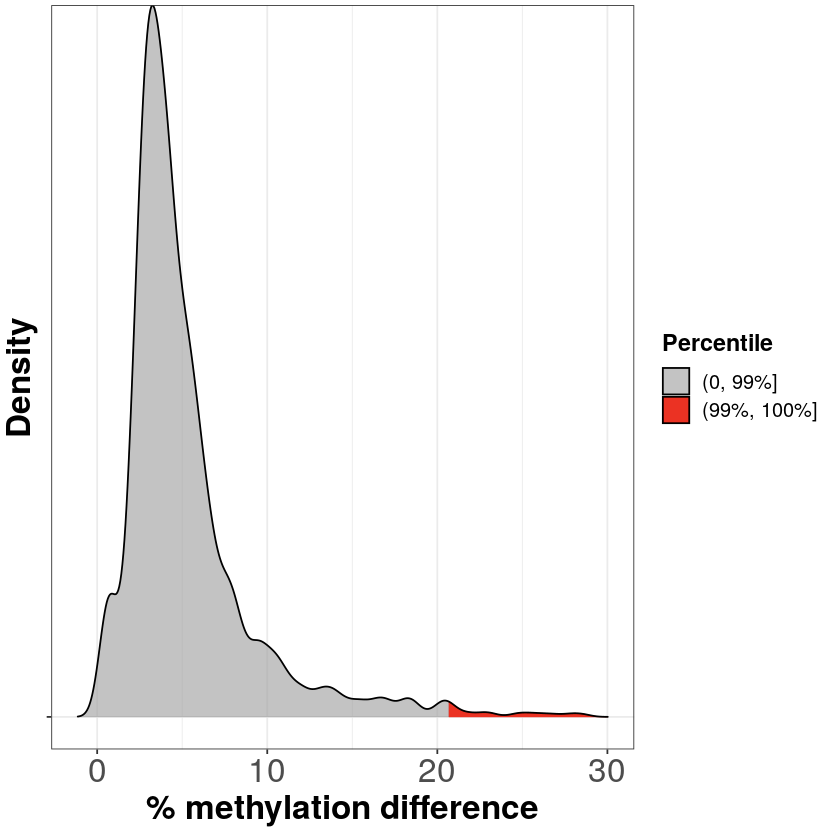


**Supplementary Figure 2:** Distribution of % methylation difference for differentially methylated loci within CpG islands exclusive to 4-month exposure groups.


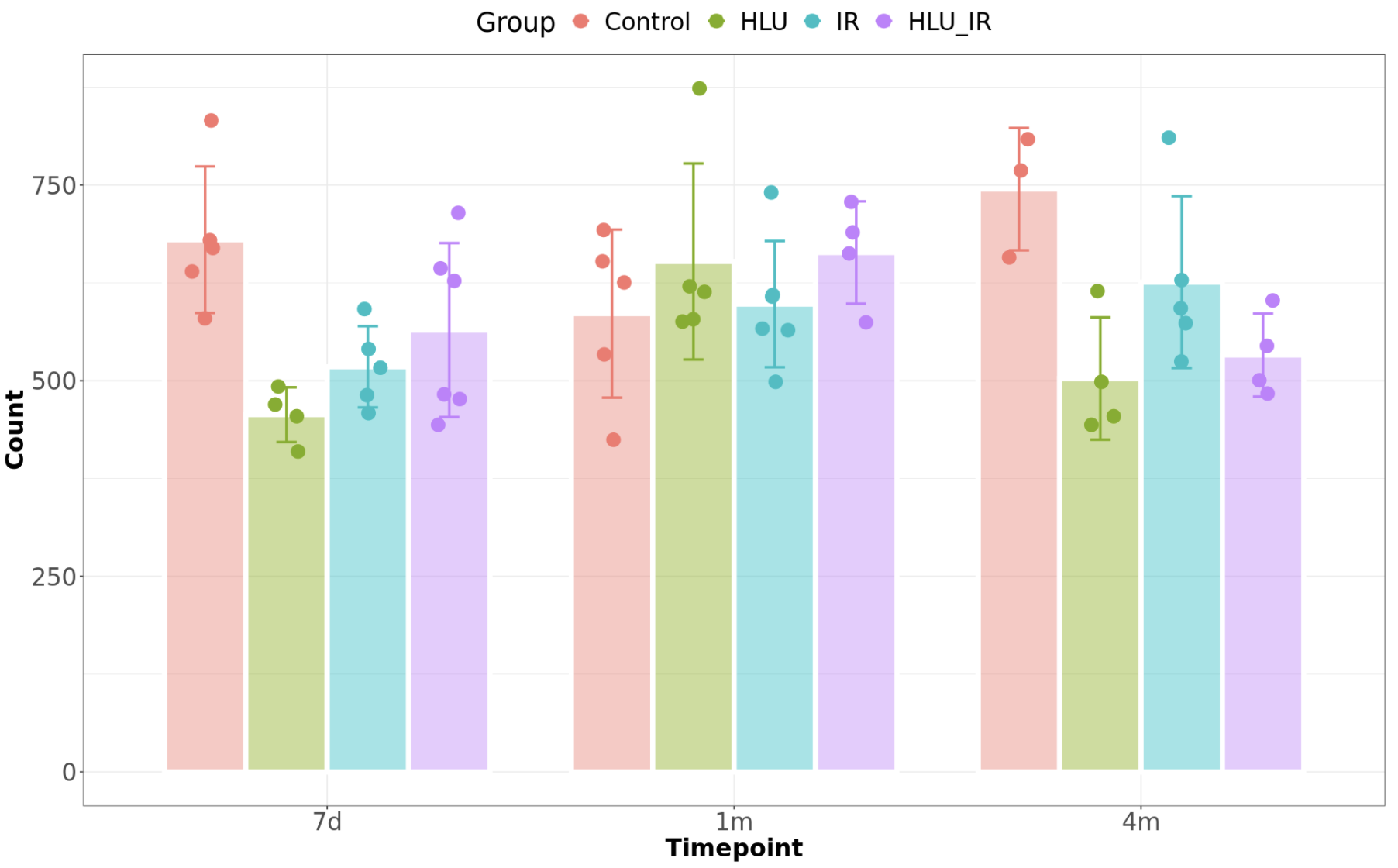


**Supplementary Figure 3:** Normalized RNA-seq counts for *Pdk1* across control and exposure groups at different time points.


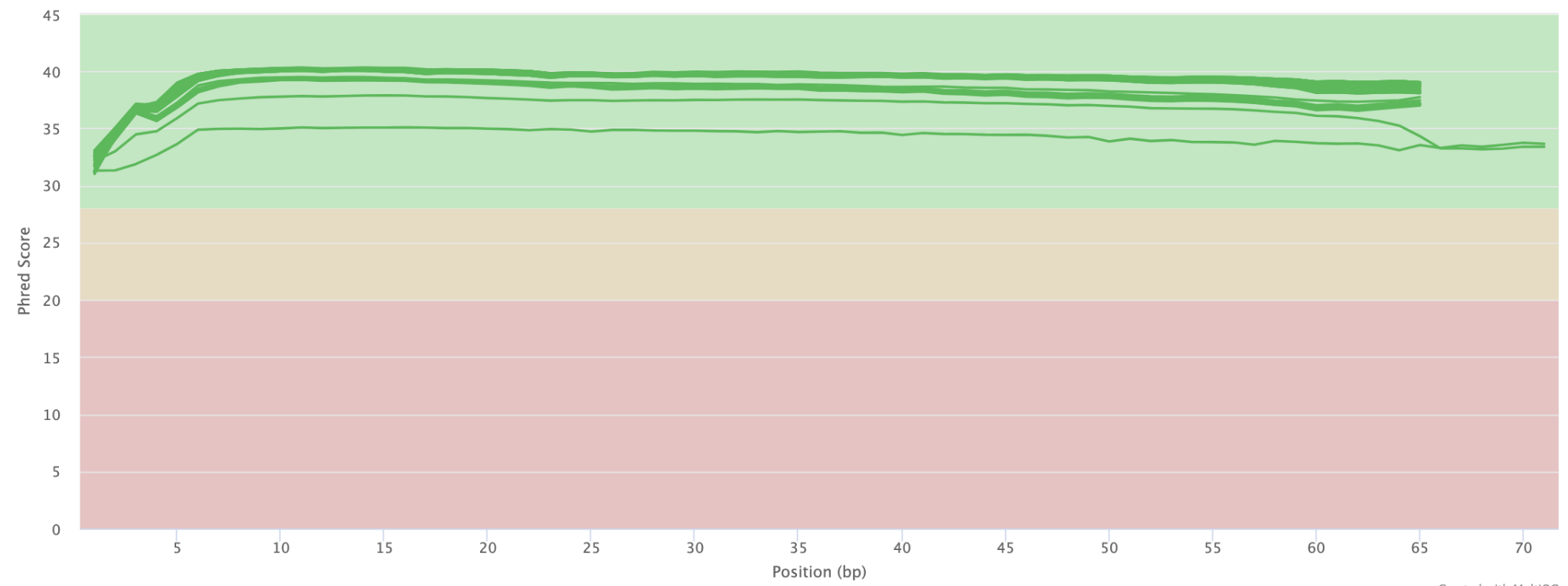


**Supplementary Figure 4**: Mean Phred quality score across each base position in the read.

**Supplementary Tables**

Supplementary Tables as provided as an Excel workbook with separate tabs for each of the tables described below:

**Supplementary Table S1**: Sample counts across different exposure groups.

**Supplementary Table S2**: Disease term enrichment from Enrichr for differentially expressed genes 7 days after exposure to radiation alone.

**Supplementary Table S3**: List of genes enriched in somatodendritic compartment (SC) and/or known to be miR-466 targets. Enrichment analysis was performed with ToppGene.

**Supplementary Table S4:** Biological processes enriched in exposure groups based on differential expression results. Gene Set Enrichment Analysis (GSEA) was performed using a list of all genes ranked by Wald test statistic from DESeq2 differential expression analysis. Significant BP at adjusted p-value < 0.05 are listed. Constituent genes, adjusted p-value and Normalized Enrichment Score (NES) are also included. ClusterProfiler was used for GSEA.

**Supplementary Table S5:** Significant biological processes enriched exclusively in each exposure condition (HLU, IR, HLU+IR) or timepoint (7d, 1m, 4m).

**Supplementary Table S6:** Biological processes over-represented in exposure groups based on differentially methylated genes. Over-representation analysis was performed using differentially methylated genes for each group, and significant BP at adjusted p-value < 0.25 are listed along with member genes and adjusted p-value. ClusterProfiler was used for over-representation analysis.

**Supplementary Table S7:** Shared biological processes enriched in exposure groups based on differential methylation and expression. Significant BP at adjusted p-value < 0.25 are listed along with adjusted p-values, member genes and associated fold-change and % methylation difference. ClusterProfiler was used for over-representation analysis.
